# Mix-and-match: an improved, fast and accessible protocol for hypocotyl micrografting of *Arabidopsis* seedlings with systemic ACC responses as a case study

**DOI:** 10.1186/s13007-022-00859-1

**Published:** 2022-03-04

**Authors:** L. Vanderstraeten, R. Sanchez-Muñoz, T. Depaepe, F. Auwelaert, D. Van Der Straeten

**Affiliations:** Laboratory of Functional Plant Biology, Department of Biology, Ghent University, K. L. Ledeganckstraat 35, Ghent, Belgium

**Keywords:** Micrografting, *Arabidopsis thaliana*, Chimeric plants, Long-distance transport, Hypocotyl

## Abstract

**Background:**

Grafting is a technique widely used in horticulture that also has been applied in agriculture. In plant physiology, grafting facilitates the elucidation of mechanisms underlying growth and developmental processes, through the construction of chimeric plants with organs of different genotypes. Despite its small size, the model species *Arabidopsis thaliana* is very amenable for grafting, which can be useful to investigate transport of nutrients, amino acids or secondary metabolites between different tissues, or to investigate developmental processes depending on root-to-shoot communication, such as shoot branching, root and shoot plasticity upon shade avoidance, or disease resistance. Nevertheless, grafting protocols are usually technically challenging and training is required to achieve a reasonable success rate. Additionally, specialized tools and equipment are often needed, such as chips to accommodate the grafted plantlets or collars to maintain the contact between root and shoot.

**Results:**

In this methodology paper, we provide a fast, easy, accessible, and specialized equipment-free protocol that enables high success ratios. Critical steps and notes are detailed, easing the implementation of the procedure for non-trained researchers. An example of the protocol application by three independent non-trained researchers shows that this method allows to achieve a 90–100% of grafting efficiency after 6 days post-grafting recovery. In addition, the grafting of Col-0 with the *acs8x* mutant, depleted in 1-aminocyclopropane-1-carboxylic acid (ACC), the biosynthetic precursor of the phytohormone ethylene, provides an example of the application of this optimized protocol, showing the suitability of the process to study long-distance transport processes.

**Conclusions:**

We present an optimized protocol for hypocotyl grafting of 4-day-old *Arabidopsis thaliana* seedlings. The combination of conditions yields a grafting success of 90–100% and provides an easy and accessible methodology, reducing the time frame, and without the necessity of acquiring specialized equipment. The presented protocol is simple, fast and highly efficient, easing the inclusion of hypocotyl grafting assays in any research project. In addition, the description of the protocol is detailed to a level ensuring that even non-trained researchers, are sufficiently prepared to adopt the grafting methodology.

## Background

Grafting is a well-established technique that has been extensively used in horticultural practices for centuries, allowing vegetative propagation of plants [24]. More recently, it has been applied in molecular research as well [30]. *Arabidopsis thaliana* micrografting has been performed for over 20 years, allowing the formation of plants with different genotypes of shoot (scion) and root (rootstock) at very early developmental stages. Grafting at a young age has several advantages, but the fact that seedlings have to quickly regenerate post-grafting is a most important point of attention. As evidenced by a rapid vascular reconnection, seedlings continue growing already after 1 week [22]. The formation of chimeric plants by means of micrografting allows an in-depth investigation of a plethora of plant developmental processes including shoot growth [34], shoot branching [5, 32], root-to-shoot signaling [10], flowering time [11, 25], shade avoidance [29], senescence [37], but also hormone signaling [36] and stress resistance [8, 9]. Grafting is also used to elucidate transport of long-distance signals both from root-to-shoot and vice versa, including the transport of small RNAs [7, 23, 26], lipids [15], and nutrients [16, 35]. During the past few years, micrografting has been also applied to study the transport of plant hormones. For example, Matsumoto-Kitano et al. [19] analysed the necessity of shoot-to-root or root-to-shoot transport of cytokinins for cambial activity. In 2009, Hayward et al. [17] showed the interplay between auxins and strigolactones by the long-distance transport of strigolactones between scions and rootstocks from different deficient mutants. Ragni et al. [27] used a combination of micrografting and gene expression analysis to investigate the role of gibberellin transport during hypocotyl xylem expansion, while Schulze et al. [28] performed micrografting experiments in combination with hormone profiling to investigate the relocation of endogenous jasmonates through the phloem after wounding.

Two different *Arabidopsis* hypocotyl-hypocotyl micrografting techniques have been developed in the past years [20, 21, 32]. The first technique makes use of silicon tubes (also known as collars) that are placed across the graft junction to assist in attachment and stabilization of the graft [3, 32]. Although this technique is quite time-consuming and requires the use of the silicon collars, it significantly reduces adventitious root (AR) formation. Formation of ARs on the scion is highly undesirable since it can replace the function of the rootstock and confound the results in downstream applications [32]. Hence, grafted plants displaying AR development have to be discarded. A second protocol makes use of a Hybond-N membrane on top of two wet filter papers onto which the seedlings are transferred pre-grafting [20, 21]. These two protocols, along with other *A. thaliana* grafting techniques were recently reviewed by Bartusch and Melnyk [4].

As stated before, regardless of its extensive application in literature, the use of grafting techniques always represents a challenge. Apart from being time-consuming and from the necessity for a large number of samples due to the potential low efficiency of the process, adopting this technique requires a steep learning curve and only after some training, the researcher is able to achieve high success ratios [4]. Furthermore, it often requires specialized equipment to improve the grafting procedure. Here we present an optimized method for the sterile hypocotyl-hypocotyl flat-surface grafting of young *Arabidopsis* seedlings. The protocol is based on the second grafting technique described above [20, 21, 32] with the optimization of pre- and post-grafting growth conditions in order to achieve higher success ratios. The previously published grafting protocols that were used as a basis for our protocol resulted in reasonably high success rates (50–70% for Ref. [3], 70% for Ref. [32] and 80% for Ref. [20] and [21]. After careful adjustment of pre-grafting growth and post-grafting recovery conditions, we were able to obtain an average of 85–90% success. In addition, the optimization of both the conditions and pre- and post-grafting incubation reduces the total duration of this protocol, increasing the number of samples that can be processed. In order to allow non-trained researchers to easily master the technique, we provide a detailed point-by-point protocol, highlighting critical steps, clarifying notes, and some guiding images.

## Results

### An example of the micrografting protocol application: Root-to-shoot communication of ACC

**Fig. 1:**
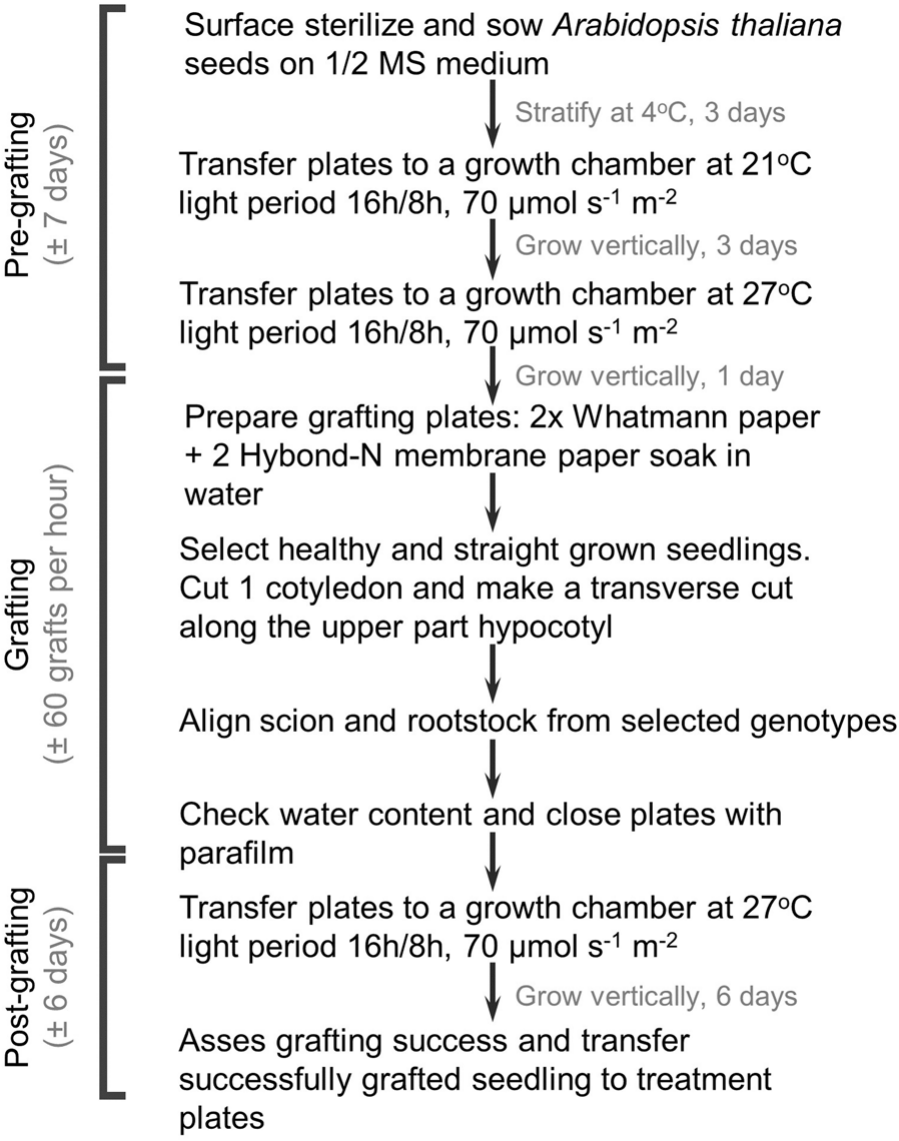
Simplified overview of *Arabidopsis thaliana* micrografting. Flow chart outlining the timeline and key steps in the process. Timing indicates time between steps

To test our optimized protocol in terms of efficiency and applicability to root-to-shoot communication, we used wild type Columbia-0 (Col-0) and the ACC synthase (ACS) octuple mutant (*acs8x*) *A. thaliana* plantlets. *acs8x* mutants are known to display a reduced rosette size due to the drastically lowered levels of ACC, the soluble biosynthetic precursor of the plant hormone ethylene, in shoot tissues [31]. ACS and ACC oxidase (ACO) are enzymes involved in ethylene biosynthesis and, therefore, their modulation has direct implications in diverse physiological processes triggered by ethylene signaling [1, 2, 13, 33]. Transport of ACC from roots to shoots upon root waterlogging was previously demonstrated in both tomato and rice [6, 14]. Here we evaluated the presence of long-distance root-to-shoot communication of ACC during vegetative growth of *A. thaliana*. *acs8x* scions were grafted onto Col-0 rootstocks, following the protocol detailed above (Fig. 1). After 6 days post-grafting, the efficiency obtained by three independent non-trained researchers reached more than 90% after only one less-efficient first attempt (Table 1) (for more details, see “Assessment of grafting success” section).

**Table 1:** Success assessment after 6 days post-grafting recovery of grafted *Arabidopsis thaliana* plantlets

	Total number of grafted seedlings	Successfully grafted (assessed after 6 days)	Unsuccesfully grafted seedlings		Percentage of success (%)
			Number of seedlings developing AR	Not aligned scion and rootstock	
First time					
Researcher 1	30	14	7	9	46.6
Researcher 2	40	21	5	14	52.5
Researcher 3	40	12	9	19	30
Second time*					
Researcher 1	29	25	1	3	86.2
Researcher 2	29	27	0	2	93.10
Researcher 3	57	54	0	3	94.7

The data were collected from grafting experiments of three independent non-trained researchers, and differentiate between the first and the second attempt

Total number of grafted seedlings is indicated, as well as both the successfully and unsuccessfully grafted seedlings (differentiating between the ones that developed adventitious roots (AR) and the ones with poor root-to-scion alignment)

The percentage of success is calculated as successfully grafted seedlings/total number of seedlings

A paired one-tailed T-test was used to identify significant differences between success rates at the first and the second attempt (*p-value > 0.05)

Successfully grafted seedlings were transferred to half-strength MS medium supplemented with 1% sucrose and solidified with 0.8% agar. Plates were transferred to a tissue culture chamber at 21 °C (16/8 h photoperiod; 70 µmol s^−1^ m^−2^) and cultured for 3 weeks in a horizontal position. After 3 weeks of growth, rosette size was evaluated by photographing the plants with a Canon EOS 550D camera (Canon, Tokyo, Japan) and measuring with Rosette Tracker [12].

**Fig. 2:**
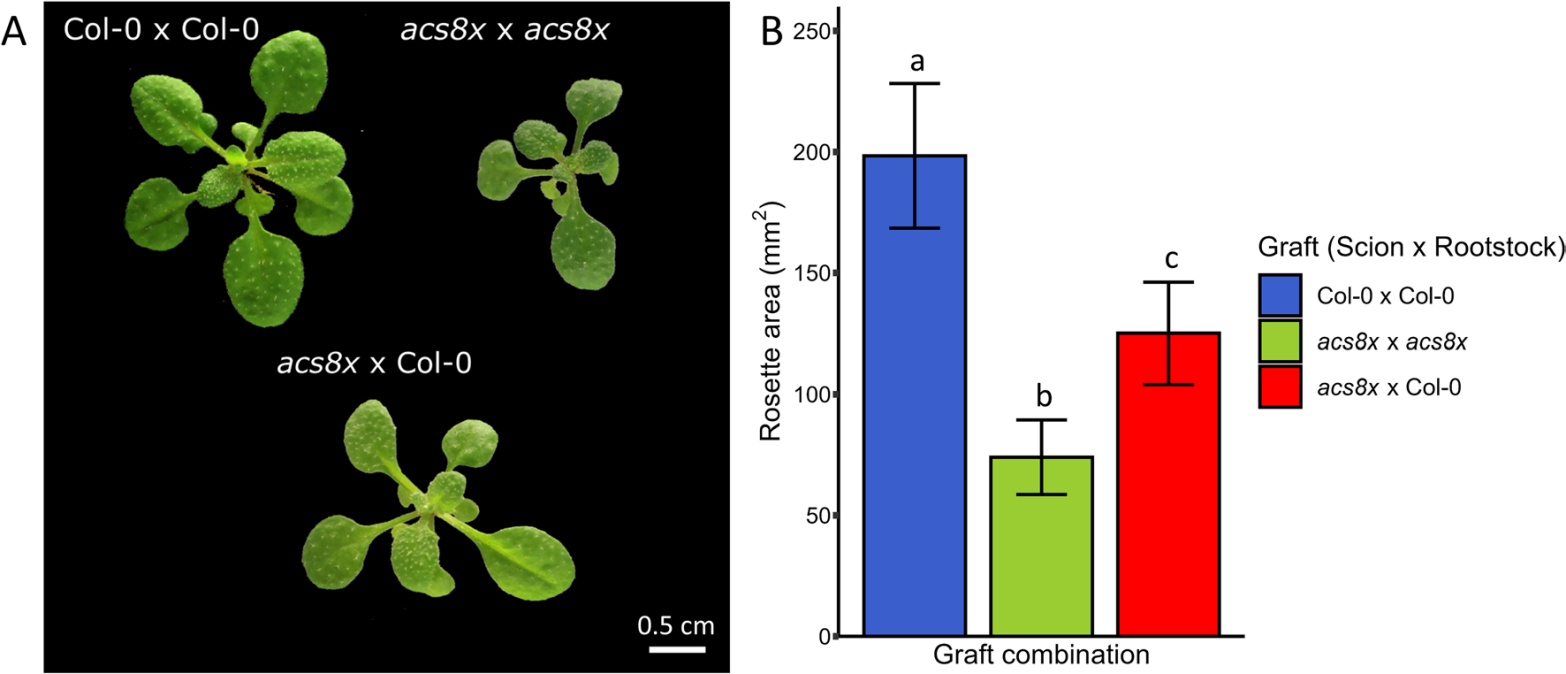
Rosette size comparison between the graft combinations: Col-0 × Col-0, *acs8x* × *acs8x* and *acs8x* × Col-0 (scion × rootstock). **A** Rosette images from the three graft combinations. **B** Rosette area (mm^2^) of Col-0 × Col-0, *acs8x* × *acs8* and *acs8x* × Col-0 graft combinations (*acs8x* is depleted in the ethylene precursor ACC). Results are expressed as the mean ± standard deviation (n = 30, 3 independent replicates). A non-parametric Krustal-Wallis test (*P* < 0.01) followed by post hoc Dunn’s Multiple Comparison Test was used (*P* < 0.01) to assess the effect of the graft combination on rosette area. Different letters represent significant statistical differences

As expected, we observed that the grafted controls Col-0 × Col-0 and *acs8x* × *acs8x* showed a significant difference in rosette size. The rosette area of the *acs8x* x *acs8x* combination was less than halfthat of the control Col-0 × Col-0 (Fig. 2A), in accordance with the rosette sizes of mutant plants in Tsuchisaka et al. [31]. Interestingly, the combination of the *acs8x* scion and the wild-type rootstock, where the roots are able to synthetize normal levels of ACC, resulted in an increase in rosette size (Fig. 2B). Thus, an *acs8x* × Col-0 graft combination resulted in a partial reversion of the *acs8x* phenotype indicating a role for ACC synthesized in roots in the regulation of rosette development. These results are in accordance with the data of Tsuchisaka et al. [31], where acs8x plantlets supplemented with different concentrations of ACC were able to show a wild type-like phenotype regarding rosette size. Accordingly, our example reveals that a root-to-shoot ACC flow is able to reverse the initial *acs8x* phenotype, confirming (1) the success of the vascular regeneration and (2) the suitability of the methodology to study long-distance transport, in this case, of the ethylene precursor ACC. Additionally, our example corroborates previous reports demonstrating that ACC can act as a mobile signal leading to ethylene responses in distant tissues [6, 14].

## Discussion

Despite its small size, *Arabidopsis thaliana* is an excellent plant species for grafting experiments. This is, in part, due to its many advantages including its short life cycle, the available genetic resources, and the rapid graft formation. In addition to the hypocotyl grafting technique for which this protocol represents an optimimalization, other grafting techniques (such as inflorescence, petiole-to-scion, root tip and epicotyl grafting, among others) have been performed in the past allowing researchers to study different aspects of plant development [4].

The success rate of seedling hypocotyl grafting is dependent on three vital steps in the grafting procedure. A first critical point is the seedling age and pre-grafting growth conditions. Some previous growth protocols suggest growing the *Arabidopsis thaliana* seedlings under short day or low light conditions [20]. These conditions result in a significantly larger hypocotyl, however, using those conditions, the pre-grafting age should be prolonged to 6–7 days as in this protocol, instead of 4 days. The second crucial step in the protocol is the grafting itself. Cutting blades should be very sharp so that the hypocotyl is effectively cut and not squished. The cutting site should be located in the upper part of the hypocotyl, preferentially at 1/4th of the length of the hypocotyl, from the shoot apex down. When the grafting is finished, water content should be inspected and adjusted if necessary. Excess water is one of the main causes for grafting failure and adventitious root formation, while too little water will dry out the seedlings. In case too much water is observed, the plates can be opened to allow evaporation until the water film has disappeared. Another possibility is to reduce the excess water using dry Hybond-N strips. The third and last crucial part of the grafting procedure is the post-grafting growth phase. Plates should be sealed correctly with parafilm to avoid drying out the plates. The plates are to be placed vertically in a growth chamber at 27 °C. This elevated temperature significantly increases grafting success rates [32]. After optimization and combination of the formerly described conditions, the grafting protocol presented here resulted in success rates of 90–100% after initial assessment and transfer to treatment plates. In the subsequent treatment period, some additional grafts were lost due to unsuccessful seedling recovery, leading to an overall grafting success of 85–90%. These success rates were higher than the previously presented protocols. The protocol form Bainbridge et al*.* [3] resulted in 50–70% grafting success. In this protocol, scion and root were held together using silicon tubings and grafts were placed on media plates. The protocol from Turnbull et al. [32] resulted in 70% grafting success. In this protocol, cellulose nitrate membranes are used instead of Hybond-N membranes and only one layer of Whatman filter paper is used. In addition, plates were incubated in growth chambers at 23 °C, with 16 h/8 h light photoperiod both pre- and post-grafting. The protocols from Melnyk et al. [20, 21] resulted in an average success rate of 80%. Our optimized protocol is most similar to these protocols, but the specific combination of conditions yields an improved final outcome that arrives to an 85–90% overall grafting success after 3 weeks growth. Specifically, the seedlings were grown on plates with 0.5% sucrose and transferred to a growth room at 27 °C, 1 day pre-grafting and 6 days post-grafting. The double layer of Whatman filter paper and the Hybond-N membrane also provide a better environment to maintain the moisture of the grafted seedlings. Apart from the high success rate, this protocol also reduces the required time frame, leading to higher as well as faster throughput and it does not require specialized equipment to perform the grafting procedure.

Finally, one of our main objectives was to offer an easy and ready-to-use grafting protocol to the plant community. With this purpose, we detailed all the steps, provided notes on the critical points, and figures of the different stages of the procedure. Since it is well known that other grafting protocols require long training times to master the techinique, the protocol presented here was tested by three independent non-expert researchers that, after only one less-efficient attempt, were able to achieve efficiencies higher than 90% of grafting efficiency on average. These results prove that the protocol reduces the necessary learning curve to a minimum, being easily accessible to non-experienced researchers.

## Conclusion

The present work provides a fast, easy, efficient and accessible methodology that will be helpful to researchers aiming to include grafting assays in their studies. The optimized success rate surely raises confidence in the obtained results upon different treatments and improves time management. With this extensively detailed protocol we prepared a ready-to-use optimized methodology that will be helpful to the scientific community. We therefore hope that this potent tool will be extensively used in plant developmental biology without any fear, since it is an elegant approach to study transport of both signals and metabolites with the final goal of unraveling complex developmental mechanisms.

## Methods

Before detailing the grafting procedure, some conditions need to be considered regarding the cutting surface, the removal of cotyledons, the light exposure, growth temperature and seedlings age.

### Grafting cutting environment

Different possibilities regarding the choice of the grafting cutting enrivonment have been reported. We have found that the double Whatman-Hybond-N membrane combination in empty small round petri dishes results in the highest grafting success, owing to the maintenance of the desired moisture level. Placing the Whatman-Hybond-N membranes in plates containing growth medium results in unwanted growth where scion and root are pushed from one another before proper graft formation. In our hands, using nitrocellulose membranes as suggested by Turnbull et al. [32] resulted in problems with moisture content. In addition, the manipulation of the seedlings in plates without medium eases the procedure, making it more manageable.

### Cotyledon removal

In this protocol, one cotyledon was removed. It helps the hypocotyl to lay flat on top of the Whatman-Hybond-N membranes allowing easier manipulation and grafting, as described by Melnyk et al. [20, 21]. Cutting both cotyledons results in a reduced growth and therefore reduced grafting success after recovery of seedlings.

### Light conditions

Seedlings were grown under long day conditions (16/8 h photoperiod; 70 µmol s^−1^ m^−2^). These conditions allow seedlings to grow faster rendering their manipulation easier. In contrast, short day conditions generate seedlings with longer hypocotyls, but they require longer growing times [20].

### Temperature

One of the key points in the regeneration of the vascular system is temperature. A pre- and post-grafting incubation at 27 °C has shown to be indispensable to induce wound healing and to avoid adventitious root growth, a signal of a deficient vascular regeneration. Incubation at 27 °C during 24 h pre-grafting, and 6 days post-grafting to induce wound recovery has been shown to drastically increase the success of the grafting [32]. For that, these two key points were included and specifically highlighted as critical points in the optimized grafting protocol.

### Time: pre- and post-grafting culture

The combination of the abovementioned conditions allows to reduce growing times, reducing the necessary time for grafting assays considerably [from 19–23 days [18] to 14–22 days [21] indicated in standard protocols to 12–13 days (counting from seed sterilization to grafting efficiency assessment)]. Specifically, seedlings were grown for 4 days prior grafting and 6 days post-grafting.

### Required materials

#### Plant material and reagents

- Sterilized *A. thaliana* seeds of the required genotype(s) (here we used wild type Col-0 and the ACC synthase (ACS) octuple mutant (*acs8x*) (obtained from NASC).
- Commercial 5% NaOCl solution (bleach; Delhaize, Belgium)
- 70% ethanol solution (denatured with Eurodenaturant, Disolol®, Chem-Lab NV)
- Distilled water
- Murashige & Skoog (MS) medium (Duchefa, The Netherlands)
- Sucrose (Merck, Belgium)
- Plant agar (Duchefa, The Netherlands)

#### Equipment

- Square petri dishes 120 mm × 120 mm (Novolab, Belgium)
- Round petri dishes Ø 90 mm (Novolab, Belgium)
- Fine forceps
- Microsurgery knives, ultra-thin blade, 15° cutting angle (Fine Science Tools catalog no. 10315–12)
- Whatman cellulose chromatography paper (3MM Chr sheets, 46 × 57 cm) cut into Ø 85 mm circles (Novolab, Belgium)
- Hybond N membrane paper cut into 20 × 60 mm pieces (Merck, Belgium)
- Parafilm (Novolab, Belgium)
- 3 M Micropore tape (Novolab, Belgium)
- Stereomicroscope (here, a Zeiss Stemi DV4 stereomicroscope was used)
- Sterile hood

#### Reagents and equipment preparation

- Pre-grafting culture medium: Half-strength MS (1/2 MS) medium supplemented with 0.5% of sucrose and solidified with 0.8% agar, pH 5.8 adjusted with KOH.
- Sterilize the cut pieces of Whatman cellulose and Hybond N membrane paper, 1L of distilled water per approximately 200–300 seedlings to graft, and fine forceps by autoclaving.

### Grafting procedure

#### Preparation pre-grafting

1. *Arabidopsis thaliana* seeds are surface sterilized for 12 min in a 5% NaOCl solution followed by 3–4 rinses in sterile distilled water. The sterilized seeds are plated on 1/2 MS medium containing 0.8% agar and 0.5% sucrose^1^ and the plates are placed in dark conditions at 4 °C to induce seed stratification.
2. After stratification for 3 days at 4 °C, plates are transferred to a tissue culture chamber at 21 °C (16/8 h photoperiod; 70 µmol s^−1^ m^−2^)^2^ for 3 days, placed in a vertical position.
  **Critical step 1:** Growing the seedlings in a vertical position ensures uniformly straight hypocotyl growth and eases posterior manipulation Melnyk et al. [20, 21].
3. Subsequently, the plates are transferred to a tissue culture chamber at 27 °C (16/8 h photoperiod; 70 µmol s^−1^ m^−2^) for 1 day maintaining the vertical position.^3^

#### Sterile grafting procedure

**Fig. 3:**
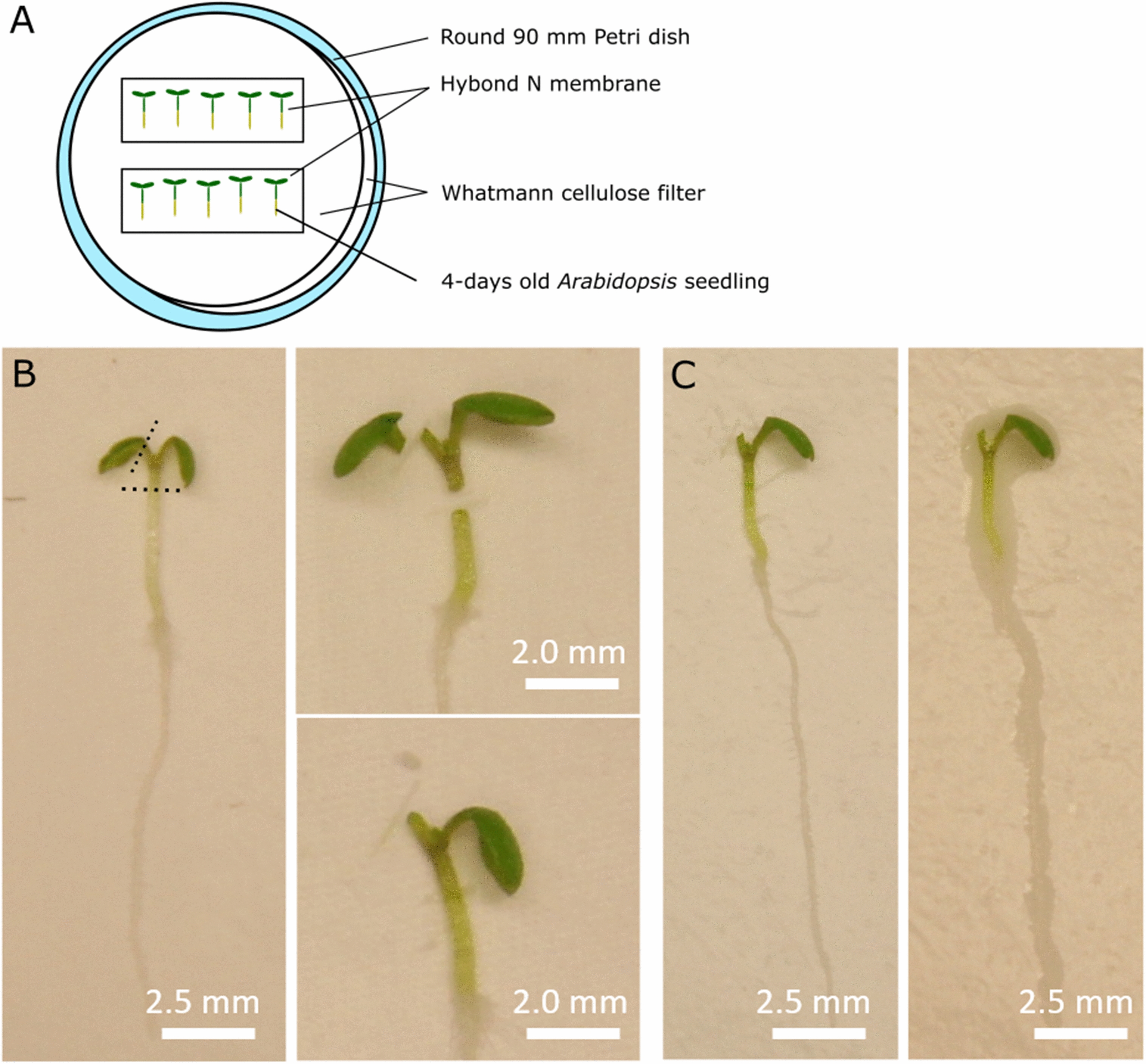
*Arabidopsis thaliana* grafting of wild type Col-0. **A** Two Whatman cellulose papers with on top two Hybond N membrane strips are soaked in sterile distilled water. They are subsequently placed in a Ø 90 mm round petri dish. Seedlings are transferred on top of the Hybond-N membrane strips. **B** Seedlings are cut and desired scion-rootstock assemblies are made. **C** When grafting is finished, moisture content is assessed. The Whatman paper and Hybond-N membrane strips should be well saturated with water (Left). A film around the seedling (Right) indicates excess water, which should be avoided around the hypocotyl

Grafting is performed under a stereomicroscope in a sterile hood. All materials used, including the forceps, the microsurgery knives and the stereomicroscope are to be kept sterile during the process using Disolol®. Sharpness of the microsurgery knifes should be assessed to allow clean cuts. If the knife does not make a straight cut or if it squishes the hypocotyl tissue, it should be discarded. In order to plan the time needed for the grafting procedure, note that this methodology allows to graft approximately 60 seedlings per hour. The detailed grafting procedure is as follows:

4. Two Whatman cellulose papers (circles of Ø 85 mm) and two strips of Hybond-N paper (20 × 60 mm) previously sterilized are soaked in sterile demineralized water and placed in a round petri dish (Ø 90 mm). The two Whatman cellulose papers should lay on top of each other, and the Hybond-N membrane strips should be placed on top of the Whatman papers as schematically shown in Fig. 3A. The combination of the two Whatman papers and the Hybond-N membranes ensures proper grafting conditions and the desired moisture content.
5. Healthy and straight grown seedlings are selected and transferred to the Hybond-N membrane. Seedlings with bended hypocotyls should be avoided since they will lead to reduced grafting success. To transfer the seedlings, seize them at one of the cotyledons with the forceps as not to damage the hypocotyl, shoot apical meristem or root. From the selected seedlings, one of the cotyledons is cut, preferably the one touched to transfer the seedlings to the plate (Fig. 3B).
6. Subsequently, a transverse cut is made across the upper part of the hypocotyl. The apical part (scion) from one seedling is then placed onto the rootstock of a second one. This can be the rootstock of the same genotype (control) or a different genotype. While transferring the scion, it is important not to damage the hypocotyl and shoot apical meristem as this will inhibit graft formation.
  **Critical step 2:** The transverse cut must be made in the first 1/4th of the hypocotyl. The higher the cut is performed in the hypocotyl, the less likely AR are formed, and thus, the higher the efficiency of grafting [32].
7. Once all grafts have been performed, moisture content of the plates is checked. The Whatman paper and Hybond membrane should be saturated with water; however, an excess of water is undesirable since it will lead to adventitious root formation (Fig. 3C). If the water content is as required, the tiny amount of water at the cutting surfaces of the scion and rootstock is sufficient to hold them together to allow proper re-establishment of the vascular connection.
  **Critical step 3:** Correct water saturation can be assessed by careful inspection of the grafted seedling. If cut parts are difficult to place and stick to the forceps, the water content is too low. If a film of excess water is seen around the seedling, the water content is too high (Fig. 3C).
8. The round petri dishes are closed and sealed with a double layer of parafilm to avoid water evaporation and ensure high humidity.

#### Post-graft culturing

9. The plates containing the grafted seedlings and the combination of two Whatman papers and two Hybond-N membrane strips are placed vertically in the 27 °C tissue culture room to allow grafts being formed by re-establishing vascular connections. The temperature of 27 °C is crucial to promote wound healing, as well as to reduce adventitious root formation.^4^
  **Critical step 4:** Growing the grafted seedlings in a vertical position ensures the contact between scion and rootstock, facilitating vascular regeneration.

### Assessment of grafting success

**Fig. 4:**
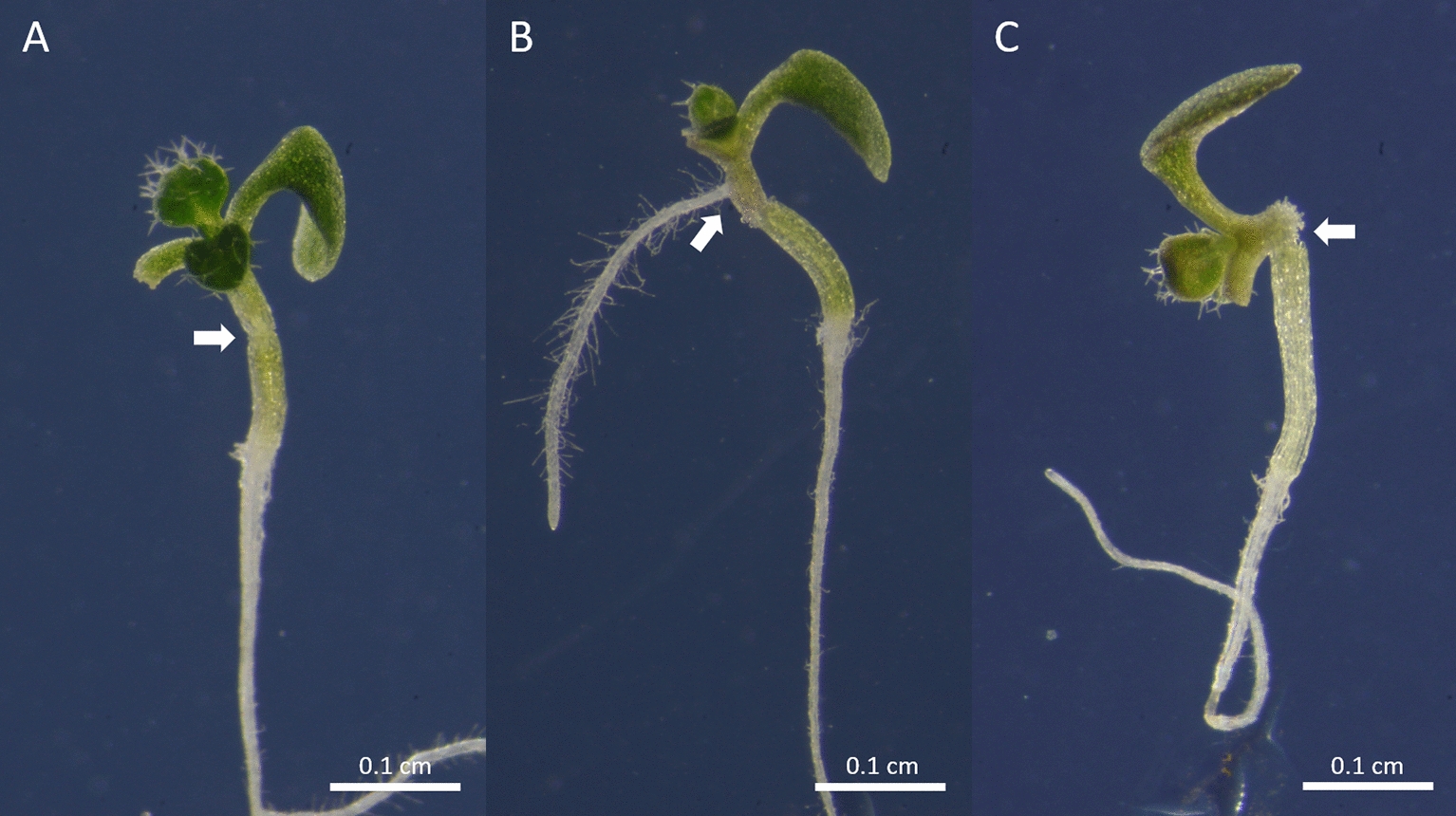
Assestment of the grafting success of *Arabidopsis thaliana* plantlets after 6 days post-grafting recovery. **A** Successfully grafted plantlet. The white arrow points toward the joint between scion and rootstock. **B** Unsuccesful grafted plantlet due to the emergence of adventicious roots, derived from a poor vascular connection (the white arrow shows the emergence point of the adventicious root) **C** Unsuccessfully grafted plantlet due to unaligned scion and rootstock. The white arrow points at the separation between scion and rootstock

**Fig. 5:**
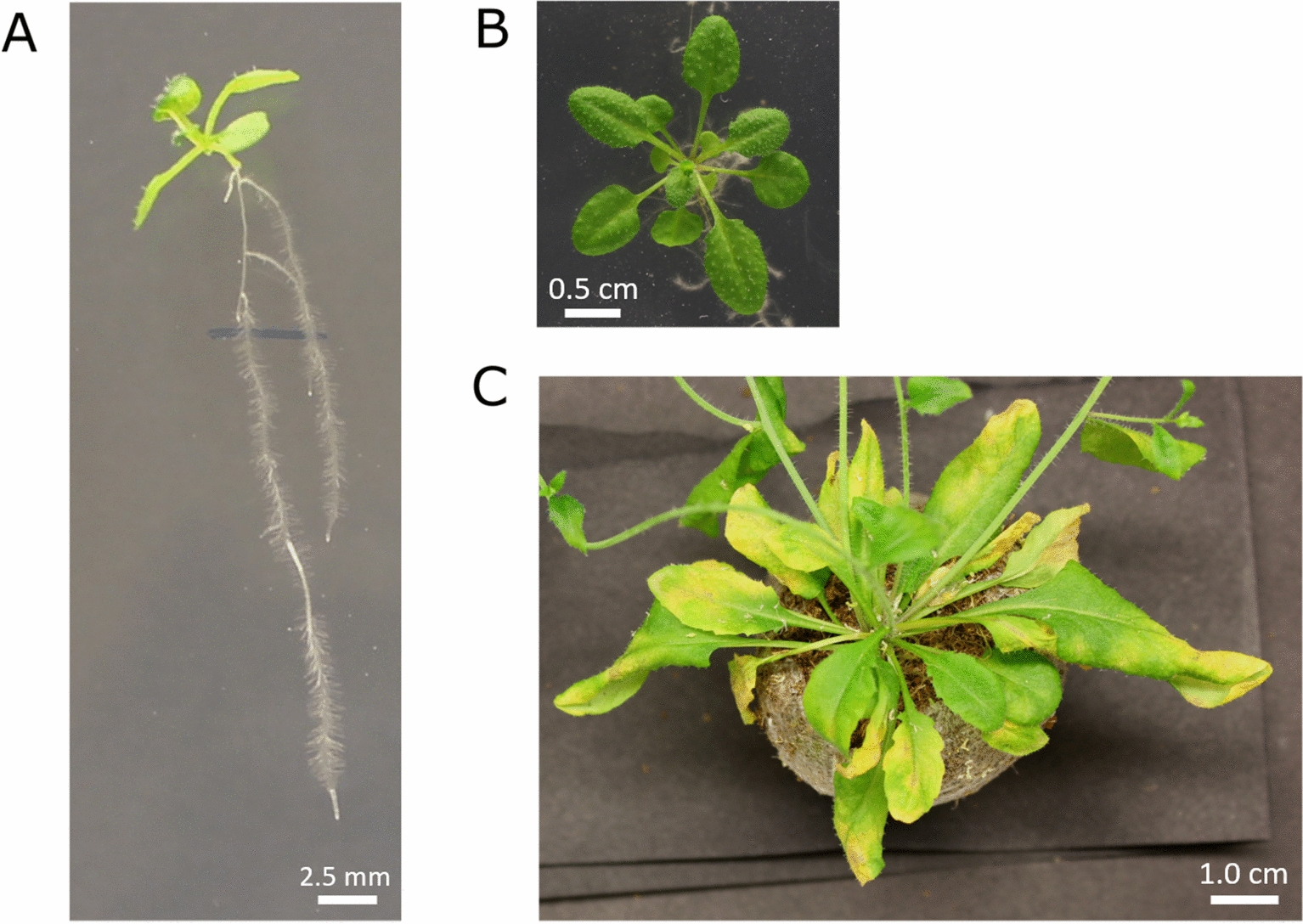
Arabidopsis thaliana grafting. Post grafting, general growth and development phenotypes can be assessed including **A** Root length analysis 2 weeks post grafting, **B** Rosette growth analysis 3 weeks post grafting, and **C** Adult senescence analysis 9 weeks post grafting

10. After 6 days post-grafting, the success of grafting is assessed under a stereomicroscope. The emergence of adventitious roots is a sign of failure of graft formation. A sign for successful graft formation is the re-establishment of root and shoot growth (Fig. 4)^5^. With this protocol, a 90–100% of grafting efficiency is obtained after 6 days post-grafting recovery.
11. Successful grafts are transferred to treatment plates (Fig. 5A, B) or soil (Fig. 5C) for phenotypic assessment depending on the desired conditions. Grafted seedlings are manipulated carefully, placing the forceps under the hypocotyl and placing them in the desired treatment plate or planting them in soil.
12. Grafted plants are continuously assessed for adventitious roots emergence and proper general development. Plants that display delayed growth due to partially or completely unsuccessful vascular reconnection, are disregarded for future assays. Here, 85–90% of efficiency representing plantlets survival after 3 weeks growth is achieved.

## Data Availability

The data generated and/or analyzed during the present study is available as supplementary information and from the corresponding author on request.

## Abbreviations

ACC: 1-Aminocyclopropane-1-carboxylic acid
ACS: ACC synthase
AR: Adventicious roots
MS: Murashige & Skoog
NACS: Nottingham Arabidopsis Stock Centre

## Footnotes

1 Supplementing the medium with sucrose helps the seedlings to grow stronger. However, if the experimental design requires a grafting experiment without the supplementation of sucrose, the seedlings should be grown for an additional day before grafting.

2 A long-day pre-grafting regime of 16 h light/8 h dark is preferential. If a short-day regime is required, the seedlings should be grown for an additional day before grafting.

3 Growing the seedlings for 1 day at 27 °C pre-grafting results in an increased hypocotyl length, allowing easier grafting.

4 Growing the seedlings at 27 °C post-grafting induces callus formation and healing, resulting in a significant increase in grafting success with reduced adventitious root formation.

5 Transferring the grafted seedlings to growth plates approximately 6 days post grafting allowed for optimal growth recovery. We found that 14 additional days of growth on half strength MS plates resulted in sufficient growth synchronicity for further phenotypic assessment.
